# Nasal Polyps in Children: The Early Origins of a Challenging Adulthood Condition

**DOI:** 10.3390/children8110997

**Published:** 2021-11-02

**Authors:** Maria E. Di Cicco, Francesca Bizzoco, Elena Morelli, Veronica Seccia, Vincenzo Ragazzo, Diego G. Peroni, Pasquale Comberiati

**Affiliations:** 1 Department of Clinical and Experimental Medicine, University of Pisa, Via Roma n. 55, 56126 Pisa, Italy; dr.francescabizzoco@gmail.com (F.B.); ele.morelli93@gmail.com (E.M.); diego.peroni@unipi.it (D.G.P.); pasquale.comberiati@gmail.com (P.C.); 2 Allergology Section, Pediatrics Unit, Pisa University Hospital, Via Roma n. 67, 56126 Pisa, Italy; 3 Otolaryngology, Audiology, and Phoniatric Operative Unit, Department of Surgical, Medical, Molecular Pathology, and Critical Care Medicine, Pisa University Hospital, Via Trivella, 56124 Pisa, Italy; veronicaseccia@gmail.com; 4 Paediatrics and Neonatology Division, Women’s and Children’s Health Department, Versilia Hospital, Via Aurelia n. 335, 55049 Lido di Camaiore, Italy; vin.ragazzo@gmail.com; 5 Department of Clinical Immunology and Allergology, I.M. Sechenov First Moscow State Medical University, 119991 Moscow, Russia

**Keywords:** adolescents, asthma, biologicals, children, chronic rhinosinusitis, dupilumab, eosinophils, nasal polyposis, type 2 inflammation

## Abstract

Nasal polyps (NPs) are benign inflammatory masses causing chronic nasal obstruction, usually associated with underlying chronic rhinosinusitis (CRS), which are rarely reported in childhood. The interest in NPs has recently increased due to new therapeutic options, namely biological agents, such as dupilumab, and an update of the European position paper on this topic was released in 2020, providing a detailed classification for these lesions and also discussing diagnostic and therapeutic approaches also in children. In childhood, NPs usually represent red flags for systemic diseases, such as cystic fibrosis and immunodeficiencies. This review outlines the recent data on NPs in childhood, focusing on predisposing factors for CRS as well as on the potential endotypes in this particular age group, for which further studies are required in order to better clarify their pathogenesis and to identify molecular biomarkers that could help achieve more personalized treatments.

## 1. Introduction

Nasal polyps (NPs) were already known at the time of the Egyptians, but their first description dates back to ancient Hindu doctors, 150 years before Hippocrates introduced the term “polyp” and described the first procedures for their removal. Over the last decade, research interest in this condition has increased due to the availability of new biological therapies, which are modifying the natural history of the disease. Many studies on the pathophysiology and management of NPs are now available, although primarily in adult populations, which have led to a recent update of the European position paper on this topic (“European Position Paper on Rhinosinusitis and Nasal Polyps 2020”—EPOS 2020; the previous version was published in 2012) [1].

NPs are relatively common in the adult population, while they are rare in children younger than 10 years old. In the pediatric population, NPs usually represent red flags indicating underlying systemic diseases, such as cystic fibrosis (CF), primary ciliary dyskinesia (PCD), and immunodeficiencies [2]. However, it should be noted that many cases of adult NPs might represent the manifestation of a process likely to have started in childhood or adolescence, similar to what is the current understanding of other common conditions in adulthood, such as chronic obstructive pulmonary disease [3]. Therefore, more research should be dedicated to address the early stages of NP development.

In this review, we sought to outline the most recent findings on pediatric NPs, particularly focusing on multiple NPs, and provide suggestions for personalized approaches for both the diagnosis and management of NPs in children.

## 2. Nasal Polyps: Definition, Epidemiology, and Diagnostic Approach

**Figure 1:**
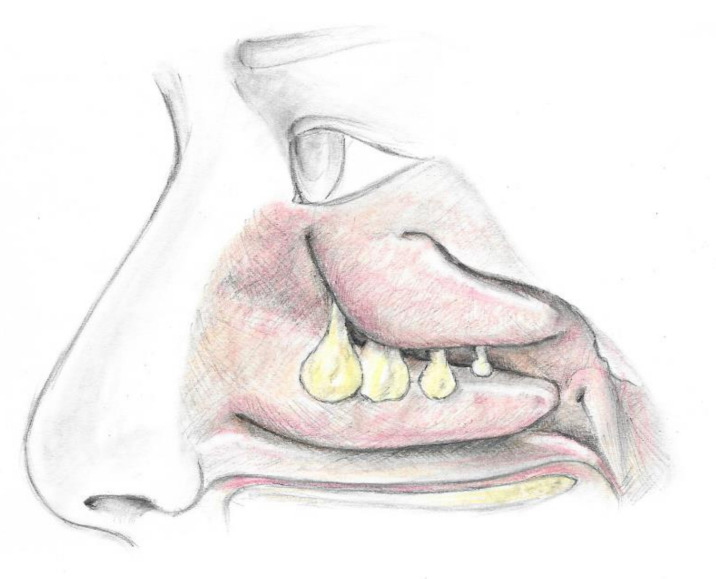
The picture shows multiple polyps arising from the middle meatus.

NPs are benign inflammatory single or multiple masses potentially arising from any portion of the mucosa of the nose and paranasal sinuses, causing chronic nasal obstruction [4,5]. They appear as translucent and gelatinous protruding digitiform/grape-like elements, with a color ranging from grayish pink to yellow, and can be easily mobilized when touched through a probe (Figure 1).

**Figure 2:**
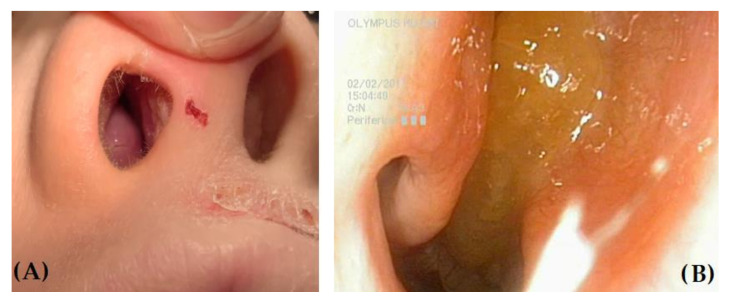
(**A**) Anterior rhinoscopy in a child with allergic rhinitis and atopic dermatitis, showing markedly hypertrophic right inferior turbinate. (**B**) Nasal polyp in the right nasal cavity: note the difference in color and morphology in the two findings.

When particularly prominent, NPs can be visualized on anterior rhinoscopy, but attention should be paid to differentiating them from other masses, such as hypertrophic edematous turbinates, which are common findings in children with severe allergic rhinitis (AR) (Figure 2).

Differential diagnosis also includes congenital defects (such as neural tube defects, dermoid cyst, meningocele, and meningoencephalocele), lymphatic tissue hypertrophy, benign tumors (nasal glioma, neurofibroma, craniopharyngioma, and juvenile nasopharyngeal angiofibroma), and malignancies (rhabdomyosarcoma, lymphoma, and nasopharyngeal carcinoma).

The most widely used classification for NPs up to 2012 was Stammberger’s [6] (Table 1), which encompasses five subgroups of polyps: the first two groups include isolated polyps, which are quite common in children, namely antrochoanal polyps, also known as Killian polyps (unilateral polyps arising from the maxillary sinus and passing into the nasal cavity to the nasopharynx) [7], and large choanal polyps (coming mainly from the contact of the two mucosal surfaces inside the ethmoidal sinuses or the sphenoethmoidal recess) [8]. The third and fourth groups include NPs associated with chronic rhinosinusitis (CRS), both with non-eosinophil-dominated and eosinophil-dominated inflammation: such polyps may be multiple and are overall rare in childhood. Lastly, the fifth group includes NPs that develop in the context of systemic diseases, such as CF and PCD.

In the EPOS 2012, a new classification focused on CRS was proposed: such condition was classified into two major phenotypes, CRS with or without NPs (CRSwNP and CRSsNP) accounting for approximately 20% and 80% of the disease, respectively; in CRSwNP, polyps must be diagnosed endoscopically as bilateral polyps in the middle meatus to avoid overlap with other polypoid diseases presenting in the nasal cavity [1].

In adults, NPs occur in 1–4% of the general population, while in children, it is a fairly rare condition, with an estimated frequency of 0.1% [2] and a significant detrimental impact on quality of life [9,10]. Epidemiological data are scarce, and in children, most of the published studies refer to children with underlying systemic diseases. In a study published in 2002, the mean estimated incidences of symptomatic NPs for all ages were 0.86 and 0.39 patients per thousand per year for males and females, respectively, with increasing rates with age (peaks of 1.68 and 0.82 patients per thousand per year for males and females, respectively, in the age group 50–59 years of age). In this study, the overall estimated incidence of symptomatic NPs was 0.627 patients per thousand per year, but, notably, no patient was under 10 years of age [11]. In a case series by Caimmi et al. [12], 56 pediatric patients with a diagnosis of NPs were reported in 17 years in a single pediatric university center in Italy. The mean age of the patients was 11.8 years, and most of them were male; 50% of the patients had a unilateral polyp and a diagnosis of antrochoanal polyp.

NP suspicion should be raised in all children with chronic nasal obstruction regardless of whether it is unilateral or bilateral or intermittent or constant [13]. In adults, an altered sense of smell and taste is also commonly reported, while in children, such disorders cannot always be assessed due to both the limited feasibility of available diagnostic tools and the delayed awareness of olfactory impairment [14,15]. Other commonly reported symptoms are posterior nasal drip, headache, snoring, and rhinorrhea. In adults, cases of massive polyposis or isolated large lesions causing obstructive sleep apnea have also been reported [1]. In general, clinical manifestations depend on the size and the location of NPs, so small-sized polyps may be asymptomatic and diagnosed by chance during rhinoscopic examinations [1].

Anterior rhinoscopy and paranasal sinus radiographs should be considered by now as outdated tests to diagnose NPs. Currently, the gold standard diagnostic test is nasal endoscopy, both in adults and children. Biopsy is rarely performed and aims to exclude other diagnoses, especially in adults with monolateral polyposis; when a biopsy is indicated, bleeding should be expected in case of high suspicion of richly vascularized tumors, so the procedure must be carried out with great care and in the best possible health care conditions. Once NPs have been detected, computed tomography (CT) may be useful for surgical planning and allows scoring the severity of CRS (the Lund–Mackay score is the most widely used, with a suggested cut-off of >5 to detect CRS in childhood) [16], but it may also overestimate abnormalities of the paranasal sinuses in the absence of or after the improvement of CRS [17]. Magnetic resonance imaging (MRI) provides superior imaging of soft tissue and is useful to detect complications and define the extension of the disease, but it is inferior for the imaging of bone [4,18].

## 3. Risk Factors for Chronic Rhinosinusitis in Children

CRS is a significant health problem affecting 5–28% of the general population and up to 2–4% of children [19,20,21].

According to the EPOS 2020 [1], CRS in children is defined as the presence for more than 12 weeks of two or more symptoms, one of which should be either nasal blockage, obstruction, congestion, or nasal discharge (anterior or posterior nasal drip), with or without facial pain or pressure and with or without cough and either endoscopic signs of nasal polyps and/or mucopurulent discharge primarily from the middle meatus and/or edema/mucosal obstruction primarily in the middle meatus and/or CT changes, such as mucosal changes within the osteomeatal complex (OMC) and/or sinuses. In these patients, cough is caused by postnasal drip and may be dry or wet, occurring at every moment of the day, while worsening at night. Such symptom has been included in the CRS definition since it represents the most common symptom in children with CRS (88%), while the most common symptom in adults is hyposmia (79%) [22,23]. As far as anterior nasal discharge is concerned, secretions may be of any quantity, and purulent or mucoid in quality, but may also be absent due to postnasal drip, which could also cause malodorous breath [24].

The lower prevalence of CRS in children is partly caused by the difficulty in assessing this condition mainly due to (i) symptoms overlapping with other common upper respiratory tract diseases and (ii) incomplete clinical examination (nasal endoscopy and/or imaging are not always available or performed in childhood), and (iii) it is a disease that is overlooked by both doctors and caregivers [1,20]. Both in adults and children, the pathogenesis of CRS arises from an alteration of the homeostasis of sinonasal drainage, which in normal conditions is warranted by the patency of the sinus ostia, the presence of an adequate and active mucous production, and an effective ciliary function. Nevertheless, children have unique risk factors for CRS when compared to adults (Table 2). In children, paranasal sinuses and their ostia are smaller and undergo a process of maturation and development, so the ethmoidal and maxillary sinuses are already present at birth, while the frontal and sphenoidal sinuses develop later, being radiologically demonstrated by the age of 4–6 [25,26], and could be at a higher risk of obstruction. Moreover, sinus disease is usually linked to the obstruction of the OMC, which is an area located in the middle meatus where secretions from the maxillary, the anterior ethmoid, and the frontal sinuses converge [27,28]. When the OMC is obstructed due to mucosal edema, negative pressure and hypoxia develop in the sinuses, stimulating mucus production and favoring its retention, which leads to bacterial growth and acute rhinosinusitis onset, giving rise to a vicious cycle further worsening the retention of secretions, impairing ciliary function, and favoring bacterial growth and biofilm formation with chronic OMC obstruction and CRS development [2]. Obstruction of the sinus ostia and OMC may be favored by allergic inflammation, the recurrence of upper respiratory infections, and adenoid hypertrophy, which are common conditions in childhood [19,23]. The latter is particularly important when considering that adenoid hypertrophy prevalence is 34% in the general pediatric population, being the most common cause for nasal obstruction in childhood, especially in younger children [29], and that due to the smaller dimensions of the upper airways in children and the proximity to the paranasal sinuses, an enlargement of the adenoids may obstruct their ostia or alter the drainage of postnasal discharge at the nasopharynx, causing posterior nasal obstruction with mucous retention. Moreover, apart from causing mechanical obstruction, the adenoids represent a reservoir for pathogenic bacteria, since their surface is potentially covered by biofilm, which may be responsible for persistent disease and the decreased efficacy of antibiotics [30,31]. Many studies have shown that in most cases of pediatric CRS, it is possible to isolate the same bacterial colonies from swabs taken from the crypts of the adenoids and from the lateral wall of the nose (mostly *Staphylococcus aureus*, *Streptococcus pneumoniae*, *Haemophilus influenzae*, and group A *streptococci*) [32,33,34]. Among other potential risk factors for CRS in childhood, tobacco smoke exposure must be underlined, since its role in CRS pathogenesis as well as in determining more severe disease and worse clinical scores is supported by many studies [35,36]. Genetic factors may also play a role in the pathogenesis of CRS [37,38].

## 4. Endotypes and Phenotypes of Chronic Rhinosinusitis with or without Nasal Polyps in Children

### 4.1. Nasal Polyps and CRS: Patterns of Inflammation

NPs are characterized by massive edema and a variable grade of inflammatory cell infiltration of the mucosa, while innervation is poor, as only sympathetic innervation can be found at the pedicle level [39]. To date, the pathogenesis of CRS and NPs has only been partially elucidated, and CRS continues to be a definition for a clinical syndrome characterized by persistent symptomatic inflammation of the nose and paranasal sinuses rather than a specific disease [1]. Historically, CRSwNP was considered as a manifestation of allergy, which was treated mostly with corticosteroids, while CRSsNP was considered to be the result of an incompletely treated acute bacterial disease, for which antibiotics were administered. However, many studies have shown that the pathogenesis of these conditions is more complex. The emerging view is that CRS is a heterogeneous syndrome with a multifactorial etiology resulting from a dysfunctional interaction between various environmental factors and the host immune system, which leads to chronic inflammation, similarly to what has been shown for asthma and the lower airways [1].

Therefore, “CRS” should be considered as an umbrella term comprising different phenotypes and endotypes with different biologic markers, types of inflammation, and interactions with the local bacterial environment, prognosis, and natural history [40]. While many researchers are trying to identify the possible triggers for inflammation at the air/mucosa interface in CRS, recent focus has been dedicated to the definition of the patterns of inflammation [41]. Currently, three different types of inflammatory patterns have been detected in adults with CRS: (i) type 1 inflammation, originally directed toward intracellular pathogens, is characterized by a predominance of IFN-γ and TNF-alpha secretion; (ii) type 2 inflammation, targeting extracellular parasites, is characterized by a predominance of IL-4, IL-5, and IL-13 secretion; and (iii) type 3 inflammation, targeting extracellular bacteria and fungi, is dominated by IL-22 and IL-17 secretion. In vivo, immunity responses are often mixed, but CRS can be defined histologically by the prevalent pattern as follows: CRS with predominant inflammation of types 1 and 3, characterized by neutrophil infiltration, and CRS with predominant type 2 inflammation, characterized by eosinophil inflammation usually with higher glucocorticosteroid response but also a high recurrence rate [42]. Chronic inflammation leads to tissue remodeling in the sinuses in a similar way to what has been described in asthma: the remodeling of the mucosa leading to the formation of polyps is characterized by a lack of TGF-ß, and an increase in metallo-proteinases, while CRSsNP is a fibrotic disease, with a surplus of TGF-ß and a high deposition of collagen [42]. The degree of tissue remodeling is greater in CRS with predominant type 2 inflammation, which is the most studied to date [43]. Notably, in Europe, 85% of NPs are characterized by type 2 inflammation, while almost the same percentage is type 2 negative and neutrophilic in Asia [44]. Consequently, eosinophils are not essential for the establishment of remodeling and NP formation. As a matter of fact, NPs in CF are as well characterized by a predominantly neutrophilic inflammation [45]. There are currently few studies on inflammatory patterns in children with CRS: nevertheless, it has been reported that both eosinophils [46] and CD4-positive lymphocytes and neutrophils [47] play an important role, with lymphocytes and neutrophils being more abundant in the sinuses of younger children [48,49,50].

### 4.2. Nasal Polyps and Allergic Diseases

The respiratory tree represents a continuum both anatomically and functionally, including the upper and lower airways, and this has recently been postulated in the so-called “global/united airway disease concept” [25,51]. Therefore, it could be speculated that conditions such as asthma, AR, and CRS are related to each other, representing different expressions of a common pathogenetic entity [52]. However, while the link between AR and asthma has been elucidated, reporting that about 40% of patients with AR have concomitant asthma and that 30–80% of asthmatics have AR, the same is not true for AR and CRS. This association is still controversial both in adults and children, with some studies showing sensitization to aeroallergens in more than 50% of children with CRS [53,54], while others have found a similar prevalence of AR in children with CRS and in the general population [55,56]. Notably, NPs are less common in people with AR than in the general population [57]. The available studies on this subject differ in terms of methodology, hampering a comprehensive evaluation; however, allergic sensitization is currently believed to play a role in the development of CRS, especially in older children, and, indeed, the EPOS 2020 suggests considering allergy testing in these patients [1]. Allergy has a pivotal role in specific phenotypes of CRS: in allergic fungal rhinosinusitis (AFRS), a severe eosinophilic polypoid CRS potentially requiring surgery and characterized by the presence of thick eosinophilic mucin with non-invasive fungal hyphae within the sinuses, hypersensitivity to fungi (such as Bipolaris, Aspergillus, Alternaria, Curvularia, and Drechslera species) is a key point [18,58]. Children with AFRS may also manifest proptosis and facial distortion and have a typical unilateral sinus involvement with non-erosive expansion [2,58]. Central compartment atopic disease (CCAD) is a subtype of adult CRSwNP associated with allergy to aeroallergens and is characterized by congestion of the anterior portion of the middle turbinate due to the recurrent exposure to inhalant allergens while breathing [59,60].

Regarding asthma, its association with CRS is supported by a huge number of epidemiological studies reporting a prevalence of asthma of about 25% in CRS compared to 5% in the general population. Around 40% of asthmatic children show radiological or endoscopic signs of rhinosinusitis [61,62], as well as the same inflammation pattern in the upper and lower airways [45]. In CRSwNP, the reported incidence of asthma ranges between 20% and 70%, and in these patients, severe asthma is common [57,63,64]. Notably, asthma shares similar endotypes with CRS, namely eosinophilic asthma (Th2-high) and neutrophilic or pauci-granulocytic asthma (Th2-low) [65]. In childhood, asthma mostly belongs to an allergic eosinophilic phenotype, characterized by a personal and/or family history of atopy and the presence of type 2 inflammation markers [66,67]. Asthma and NPs are also associated in Samter/Widal’s triad, which includes asthma, severe eosinophilic CRSwNP, and allergy to non-steroidal anti-inflammatory drugs (NSAIDs) [68]. The prevalence of the triad, currently known as NSAID-exacerbated respiratory disease (N-ERD), among adult patients with CRSwNP is reported as 16% [64], but this condition is not a prerogative of adulthood, since it has been reported in adolescents and children as young as 7 years old, so the diagnosis of N-ERD should also be suspected in this age group in the case of severe NPs with frequent need for sinus surgery [69]. The diagnosis of N-ERD is usually based on the patient history of at least one documented reaction to aspirin or other NSAIDs, but in children, this may be missing since aspirin is not recommended because of the risk of Reye’s syndrome. Aspirin provocation tests (oral, bronchial, or nasal provocation) may be needed when the history is not clear [70].

### 4.3. Nasal Polyps Associated with Chronic Diseases

Children with CF, PCD, and immunologic disorders together account for <20% of the CRS population [56]. Nevertheless, CF is the most common cause of NPs in the pediatric population: almost every CF patient suffers from CRS, and polyps begin to develop around the age of 5 [71,72,73]. Therefore, CF should be excluded in any child presenting with CRSwNP. Similarly, PCD should also be considered in the differential diagnosis of these children. PCD is a genetically heterogeneous disorder, characterized by altered ciliary structure and/or function, leading to different patterns of ciliary alteration and clinical impairment, including chronic sinopulmonary disease, laterality defects, and persistent middle ear effusion. Similar to CF, in PCD, CRS develops in childhood and lasts through adulthood, so virtually all PCD patients demonstrate pansinusitis on CT scans, and NPs are reported in 10% of children and 75% of adults [74]. These patients also frequently present frontal and/or sphenoidal sinus aplasia or hypoplasia [75,76]. As far as immunodeficiencies are concerned, the most frequent conditions causing CRS are common variable immunodeficiency (10% cases) and selective immunoglobulin A deficiency (6% cases) [77]. In children and adolescents with chronic/recurrent CRS with or without NPs, serum immunoglobulin levels should be evaluated: when suspicion is high, an evaluation of serum-specific antibody titers in response to vaccine antigens may also be considered, together with referral to a clinical immunologist [78,79]. It should be noted that patients with humoral immune deficiencies may have low or absent IgE; therefore, this finding should suggest further immunological evaluation [1].

Last but not least, ANCA-associated vasculitis, such as granulomatosis with polyangiitis (GPA, previously known as Wegener’s granulomatosis) and eosinophilic granulomatosis with polyangiitis (EGPA, previously known as Churg–Strauss syndrome), frequently and severely affect the upper airways and paranasal sinuses. GPA is a severe vasculitis affecting small-to-medium-sized vessels, characterized by granulomatous inflammation, mainly involving the airways and kidney. The majority of patients with GPA initially present with rhinological symptoms, including crusting, nasal stuffiness, discharge, and bleeding [80]. Diagnosis is usually confirmed with a positive c-ANCA test. EGPA is a necrotizing vasculitis of small-to-medium-sized vessels, associated with eosinophilia: this condition is characterized by eosinophilic granulomatous infiltration of tissues causing severe asthma and CRSwNP; p-ANCA-positivity is found in about 50% of patients [81]. The pathogenetic process of these conditions may start in adolescence, and, therefore, they should be considered in adolescents with severe CRS with or without NPs.

## 5. Treatment of Nasal Polyps: The New Era of Biological Agents

Appropriate medical therapy with nasal irrigation with saline and intranasal corticosteroids is the first-line medical treatment both in pediatric and adult CRS with or without NPs [1,82], while antibiotics and systemic steroids are used only in case of exacerbation; there is a lack of supportive evidence for prolonged macrolide therapy in children with CRS [1]. Many studies have demonstrated that topical corticosteroids are a beneficial treatment for adult CRSwNP in terms of symptoms improvement, polyp size reduction, and polyp recurrence prevention after surgery, with rare and minor side effects (the most common are epistaxis and nasal irritation) [83]. Even if no randomized controlled trial has been performed to date to support the efficacy of intranasal steroids in children affected by CRS, EPOS 2020 supports their use in light of their anti-inflammatory effects and excellent safety profile in childhood [1]. As a matter of fact, they are widely and effectively used in children with AR, confirming that the systemic bioavailability of second-generation compounds, such as mometasone and fluticasone, is less than 1%, making them safe for long-term use [84]. In younger children refractory to appropriate medical therapy, adenoidectomy is considered the first-line surgical intervention [1,28,85], followed by functional endoscopic sinus surgery aimed at reestablishing adequate sinus ventilation and secretion drainage [86,87].

Due to increasing knowledge on the inflammatory endotypes in asthma and CRS, biological treatments have recently become available. In particular, dupilumab, a fully human mAb directed toward the alpha chain of the IL-4 receptor used by both IL-4 and IL-13, is the first and only biological agent approved for the treatment of CRSwNP in adults with type 2 inflammation, regardless of the presence of asthma as a comorbidity, due to its effectiveness in reducing polyp size, sinus opacification, and symptom severity [45,64,88]. Dupilumab is also approved for the treatment of severe type 2 asthma, starting from 12 years of age [89]. There is still limited evidence on the efficacy of omalizumab (an anti-IgE) and mepolizumab (anti-IL-5) in CRSwNP, mainly in adult patients with comorbid asthma [90,91]; both of these drugs are currently approved for children aged ≥6 years with severe asthma. To our knowledge, studies on the efficacy of biologicals in CRSwNP in children are still lacking.

## 6. Conclusions

The presence of NPs is quite a rare condition in children. Nevertheless, NPs should always be suspected in children referred for chronic nasal obstruction, either unilateral or bilateral. NPs must prompt further evaluation to identify potential associated systemic conditions or predisposing factors. The treatment is based on nasal irrigation and intranasal corticosteroids, followed by surgery in non-responders. Further studies are needed to better clarify the underlying mechanisms in CRS and NPs and their endotypes, especially in children and adolescents, in order to identify molecular biomarkers and make more personalized approaches available for this age group. Even if CRS and NPs seem to be different entities in children and adults, in many adult cases, the onset is before adulthood; therefore, we should start looking for their early origins in childhood. A multidisciplinary approach is highly suggested in order to appropriately manage children with CRS and NPs.

## Figures and Tables

**Table 1:** Stammberger’s classification of nasal polyps.

Type	Systemic Diseases	Characteristics
I	Antrochoanal polyps	Isolated unilateral polyps arising from the maxillary sinus and passing into the nasal cavity and, through the posterior nostril, to the nasopharynx.
II	Large isolated choanal polyps	Large polyps in the nasal cavity, coming mainly from the contact of the two mucosal surfaces inside the ethmoidal sinuses or the sphenoethmoidal recess.
III	Polyps associated with CRS, non-eosinophil dominated	Usually bilateral, rare in children.
IV	Polyps associated with CRS, eosinophil dominated	Includes specific conditions, such as non-allergic rhinitis, Samter’s triad, and allergic fungal rhinosinusitis.
V	Polyps associated with specific diseases (CF, PCD, malignancy)	NPs developing in the context of systemic diseases.

**Table 2:** Potential and well-known risk factors for CRS in childhood.

Inflammation/Infections	Systemic Diseases	Local Factors
Recurrent upper respiratory tract infections	Cystic fibrosis	Small sinus ostia
Developing immune system	Primary ciliary dyskinesia	Anatomic variations
Adenoid hypertrophy	Immunodeficiencies	Traumas
Allergic and non-allergic rhinitis	Vasculitis	Foreign bodies
Tobacco smoke exposure		
Environmental pollution		
Gastro-esophageal reflux disease		

## Data Availability

Not applicable.
